# Comparison of the effectiveness of open, laparoscopic, and robotic-assisted radical prostatectomies based on complication rates: a retrospective observational study with administrative data from Switzerland

**DOI:** 10.1186/s12894-024-01597-3

**Published:** 2024-10-07

**Authors:** Christine von Ahlen, Alexander Geissler, Justus Vogel

**Affiliations:** 1 Technische Universität Berlin (WHO Collaborating Centre for Health Systems Research and Management), Berlin, Germany; 2 Spital Männedorf AG/Zürich, Männedorf, Switzerland; 3 Chair of Health Economics, Policy and Management, School of Medicine, University of St.Gallen, St. Jakob-Strasse 21, St. Gallen, 9000 Switzerland

**Keywords:** Radical prostatectomy, Prostate cancer, Robot-assisted, Complications, Real-world data

## Abstract

**Background:**

Radical prostatectomies can be performed using open retropubic, laparoscopic, or robot-assisted laparoscopic surgery. The literature shows that short-term outcomes (in particular, inpatient complications) differ depending on the type of procedure. To date, these differences have only been examined and confirmed in isolated cases based on national routine data.

**Methods:**

The data was based on the Swiss Medical Statistics from 2016 to 2018 from a national survey of administrative data from all Swiss hospitals. Cases with the coded main diseases neoplasm of the prostate (ICD C61) and the main treatments of laparoscopic (CHOP 60.5X.20) or retropubic (CHOP 60.5X.30) radical prostatectomies were included, resulting in a total sample size of 8,593 cases.

**Results:**

A procedure-related complication occurred in 998 cases (11.6%). By surgical procedure, complication rates were 10.1% for robotic-assisted laparoscopic radical prostatectomy 9.0% for conventional laparoscopic radical prostatectomy and 17.1% for open retropubic radical prostatectomy (*p* < 0.001). Conventional and robotic-assisted laparoscopic radical prostatectomies had a significantly lower risk of complications than retropubic procedures. Moreover, the risk of a procedure-related complication was almost twice as high in cases operated on retropubically; however, no significant difference was found between conventional and robotic-assisted laparoscopic cases.

**Discussion:**

The use of a surgical robot showed no advantages in radical prostatectomies regarding procedure-related during the hospital stay. However, both conventional and robotic-assisted laparoscopically operated radical prostatectomies show better results than open retropubic procedures. Further studies on the long-term course of patients based on claims data are needed to confirm the inherent benefits of surgical robots in tandem with them being increasingly employed in hospitals.

**Supplementary Information:**

The online version contains supplementary material available at 10.1186/s12894-024-01597-3.

## Introduction

Around 10% of all hospitalized patients worldwide have complications, half of which are preventable [1]. Moreover, the choice of the surgical technique can influence the risk of further issues in the future; for example, minimally invasive surgical procedures are less stressful for patients and have fewer complications [2]. Laparoscopic surgery is an example of a form of minimally invasive surgery that has been increasingly supported by robotics over the last ten years [3]. The advantages of robotics include three-dimensional optics, articulated instrument movements, and a filtered hand tremor [4, 5]; however, the use of robotic systems is also associated with high investment and maintenance costs which can only be covered with a high case volume [6]. Patients are increasingly seeking robot-assisted surgery, with the availability of a surgical robot often influencing the patient’s choice of hospital [7]. In addition, hospitals are investing more heavily in surgical robots to maintain their appeal to patients and specialists [8, 9].

According to the World Health Organization (WHO), around 1.4 million new cases of prostate cancer were diagnosed worldwide in 2020; this corresponds to around 7.3% of all newly diagnosed cases of cancer in men [10]. Radical prostatectomy is an important form of treatment for this disease [11], with a clear trend of hospitals transitioning away from retropubic radical prostatectomies (RRP) towards laparoscopic radical prostatectomies (LRP) and robot-assisted laparoscopic radical prostatectomies (RARP) [12, 13]. Various studies indicate that RARP is superior to RRP in terms of intraoperative adverse events, postoperative complications, and length of hospital stay [14, 15]. According to the literature, almost 90% of all radical prostatectomies in the United States are robot-assisted [16]; in the UK, this figure was 88% between 2017 and 2019 [17]. The choice of surgical procedure is typically related to individual patient factors, the surgeon’s experience, and the availability of a surgical robot [18, 19]. According to a study by Vickers et al. [20], the surgical results of radical prostatectomies improve in relation to the experience of the surgeon; however, the learning curve of surgeons is significantly flatter with LRP than with RARP. Moreover, surgeons typically require longer timeframes to learn the appropriate surgical techniques if the operation is not robot-assisted [21]. Acknowledging that the effectiveness of different types of interventions has thus far largely been evaluated via clinical trials, it is appropriate to extend the research to real-world data. While clinical studies are usually limited to the specific population and conditions of each study, administrative and billing data reflect a more comprehensive and realistic sample of the patient population, increasing the generalizability of results [22, 23]. This form of real-world data is particularly suitable for evaluating complications during hospital treatment as standardized and mandatory national regulations apply to data collection [24].

Considering the increasing number of robot-assisted operations, this study compares the complication rates after RRP, LRP, and RARP. As a first step, we identify the 25 most common procedure-related complications during hospitalization after radical prostatectomy to analyze the influence of the type of surgery on the occurrence of these complications. For this purpose, data from the Swiss Medical Statistics are used (containing the administrative data of all Swiss hospitals) which are subsequently analyzed descriptively and with binary logistic regression.

## Methods

The Medical Statistics dataset of the Swiss Federal Statistical Office is generally accessible and can be made available by the Federal Statistical Office on request. We used the years 2016 to 2018 from this dataset for our study. It contains case-level information on age, nationality, diagnosis (main and secondary diagnoses), treatments carried out, length of stay, and other administrative key figures for all inpatient cases in Switzerland.

To include all conventional or robot-assisted laparoscopic and retropubic radical prostatectomies performed in Switzerland, these were identified and classified using the Swiss surgical classification (CHOP) and the International Statistical Classification of Diseases and Related Health Problems (ICD) version ICD-10-GM 2018 [25, 26]. A radical prostatectomy for a malignant neoplasm of the prostate (ICD C61) was performed on 8,851 male patients between 2016 and 2018. For our analysis, we included 8,593 cases operated on laparoscopically (CHOP 60.5X.20) or retropubically (CHOP 60.5X.30). Excluded are 258 patients who underwent perineal (CHOP 60.5X.20) or other (CHOP 60.5X.99) radical prostatectomies. The use of a surgical robot was identified via the corresponding surgical classification (CHOP 00.99.50), which must be coded as an addition (but is not relevant for billing).

First, complications associated with the procedure were determined using the ICD codes Y69 (adverse events during surgery and medical treatment); Y84.9 (adverse events due to medical procedures, unspecified); and Y82.8 (adverse events due to medical devices and products). According to Swiss coding guidelines, these codes are additionally coded for the diagnosis that represents a complication [24]. Second, the sample was analyzed for the 25 most common procedure-related complications; all less frequently occurring complications (and complications not directly related to the procedure performed) were summarized as ‘other complications’. These were only analyzed descriptively and were not included in the logistic regression analysis; moreover, peritoneal adhesions (ICD code K66.0) were excluded as it was unclear whether these were acquired during or before the observed hospital stay. In Switzerland, complications can lead to an increase in case complexity and thus to higher remuneration; consequently, there is an incentive to fully document and code complications.

Logistic regression analysis is used to investigate whether the surgical procedure has a significant influence on the occurrence of a procedure-related complication. The dependent binary variable is the occurrence of at least one of the 25 most common procedure-related complications while the intervention types RARP and RRP are used as binary independent variables (base case: LRP). Control variables represented the age of the patients (calculation of the average within 5-year steps), the nationality (Swiss citizenship yes/no), and the insurance class (supplementary insurance yes/no). To account for comorbidities of individual cases, the comorbidity measure developed by Elixhauser et al. [27] was used as a further control variable. This measure consists of 31 weighted comorbidities and indicates how ill a patient is (the higher the value, the greater the probability of dying in hospital). This study used the weighting model for the Elixhauser comorbidity measure developed for Switzerland by Sharma et al. [28]. Lastly, we included a dummy variable indicating whether a patient received a regional lymphadenectomy as a secondary treatment, e.g., to account for its potential effects on bleeding occurrence. All analyses were performed with the statistical program SPSS.

## Results

### Descriptive statistics

In total, 3,032 (35%) of 8,593 cases were operated on using robot-assisted laparoscopy, 3,181 (37%) using conventional laparoscopy, and 2,380 (28%) using retropubic surgery (see Table 1). Roughly 40% of the cases in our sample had supplementary insurance (semi-private or private). Swiss citizenship was documented in 7,569 cases (88.1%).Patients were on average 65.6 years old and had an average length of stay of 6.7 days. The average Elixhauser comorbidity score for all cases was 0.90; this comorbidity score was higher for cases undergoing robot-assisted laparoscopic surgery (with an average value of 1.35) than cases undergoing conventional laparoscopic (0.23) and robot-assisted laparoscopic (1.22) surgery (*p* < 0.001). Thirty-five cases (0.4%) were readmitted to a hospital within 18 days due to a complication [29], and two patients died during hospitalization. Overall, at least one of the 25 most common procedure-related complications occurred in 998 (11.6%) cases while at least one other complication occurred in 280 (3.3%) cases. The number of procedure-related complications per case is shown in Appendix 1.

The two most common complications were bleeding and hematoma (234 cases, or 2.7%) and acute bleeding anemia (228 cases, or 2.7%). At 5.5%, the complication rate for retropubic surgery cases with acute bleeding anemia was significantly higher than with robot-assisted procedures (1.7%) and conventional laparoscopic (1.4%) cases. Other complications related to injuries in the surgical area and other unintended consequences of the procedure itself. The number and proportion of complications investigated for each type of procedure are detailed in Appendix 2.

**Table 1:** Descriptive statistics of the study population

Variables	Complete study	Robot-assisted laparoscopic radical Prostatectomy	Conventional laparoscopic radical Prostatectomy	Open retropubic radical prostatectomy	*P*-value
Number of cases (share of total sample)	8’593 (100%)	3’032 (35.3%)	3’181 (37.0%)	2’380 (27.7%)	-
Cases with supplementary insurance (share per surgical procedure)	3’448 (40.1%)	1’206 (39.8%)	1’525 (47.9%)	717 (30.1%)	< 0.001
Cases with Swiss citizenship (proportion per surgical procedure)	7’569 (88.1%)	2’631 (86.8%)	2’780 (87.4%)	2’158 (90.7%)	< 0.001
Average age (standard deviation per surgical procedure)	65.6 (6.8)	65.0 (6.7)	65.4 (6.8)	66.6 (6.8)	< 0.001
Average length of stay (standard deviation per surgical procedure)	6.7 (3.6)	6.0 (3.9)	6.2 (3.0)	8.2 (3.3)	< 0.001
Average Elixhauser Comorbidity Measure (standard deviation per surgical procedure)	0.90 (5.8)	1.35 (5.99)	0.23 (5.10)	1.22 (6.4)	< 0.001
Reentry due to complication (proportion per surgical procedure)	35 (0.4%)	10 (0.3%)	15 (0.5%)	10 (0.4%)	0.676
Cases died during hospitalisation (proportion per surgical procedure)	2 (0.02%)	1 (0.07%)	0	1 (0.08%)	0.681
Cases with regional lymphadenectomy as a secondary treatment	5’329 (62.0%)	1’899 (62.6%)	1’969 (61.9%)	1’461 (61.4%)	0.614
Number of cases with at least one of the 25 most frequent procedure-related complications (proportion per surgical procedure)	998 (11.6%)	306 (10.1%)	285 (9.0%)	407 (17.1%)	< 0.001
Cases with other complication (share per surgical procedure)	280 (3.3%)	94 (3.1%)	95 (3.0%)	91 (3.8%)	0.183

Note: The *p*-values for continuous variables were calculated using ANOVA, and for categorical variables using the Chi-square test

### Model results

Table 2 shows the results of our model. The regression coefficient for RARP was slightly positive (0.088) but not statistically significant (*p* = 0.312); thus, no statistically significant difference in the risk of complications (compared to conventional laparoscopic radical prostatectomies) could be observed. In contrast, retropubic surgery with a regression coefficient of 0.671 showed a significant correlation (*p* < 0.001) between this surgical procedure and the occurrence of complications compared with conventional laparoscopic surgery. Focusing on the odds ratio for RRP (1.957), it can be seen that the probability of a complication is almost twice as high with the retropubic surgical procedure compared with conventional laparoscopic surgery. In addition, patient age and the Elixhauser comorbidity measure had a significant influence on the occurrence of complications with regression coefficients of 0.022 and 0.036, respectively (*p* < 0.001).

**Table 2:** Model results of the examination of individual variables in relation to the occurrence of procedure-related complications

*Dependent variable: Presence of at least one procedure-related complication of radical prostatectomy*				
	Coeff	SE	p	Odds Ratio
Robot-assisted laparoscopic surgical procedure	0.088	0.087	0.312	1.092
Open retropubic surgical procedure	0.671	0.084	0.001***	1.957
Existence of a supplementary insurance	-0.045	0.072	0.534	0.956
Nationality: Swiss	-0.088	0.107	0.410	0.916
Age	0.022	0.005	0.001***	1.022
Elixhauser Comorbidity Measure	0.036	0.005	0.001***	1.037
Regional lymphadenectomy as a secondary treatment	0.062	0.071	0.382	1.064

Model summary: Nagelkerkes R-Quadrat: 0.036

Notes: Coeff: Estimation coefficient; SE Standard Error; *** *p* < 0.001; ** *p* < 0.01; * *p* < 0.05 The value of 0.033 for Nagelkerker’s R-squared shows that the independent variables in the present model explain some of the variability in the occurrence of an intervention-related complication. This is due to overall complication rates being low. In addition, the model is not intended to predict complications but to analyze whether there is a difference between the surgical procedures

## Discussion

In our study, we used a national data set from Switzerland to analyze the influence of different surgical procedures for radical prostatectomy for prostate cancer on the occurrence of procedure-related complications during hospital stays. Since real-world data was analyzed, the results are particularly robust, showing an almost doubled risk of procedure-related complications when retropubic surgery was performed (compared with laparoscopic surgery). Procedure-related complications occurred to a similar extent with RARP (10.1%) and RLP (9.0%) but were less frequent than with RRP (17.1%). In addition, the results showed that laparoscopically performed radical prostatectomies significantly reduced the likelihood of procedure-related complications during hospitalization, albeit with no significant difference between robot-assisted laparoscopic and conventional laparoscopic surgery.

### Classification of the results in the literature

Our results showed that laparoscopic surgical procedures are superior to retropubic surgical procedures in terms of procedure-related complications overall; however, they also showed that quality gain could not be increased by utilizing a surgical robot. De Carlo et al. [15] came to the same conclusion when they compared the three different types of surgery in a systematic review. Moreover, a study by Ilic et al. [30] reported no high-quality evidence for better oncological results and presented similar results for severe post-operative complication rates, sexual quality of life, and urination. In addition, Coughlin et al. [31] justified the advantages of the robot-assisted procedure solely based on its minimally invasive nature. Meanwhile, with an overall complication rate of 12.3% in RARP, Moretti et al. [18] showed similar results to the present study, concluding that the laparoscopic surgical procedures were beneficial for patients, particularly with the use of a surgical robot. Overall, our findings confirm the present state of the literature and add two main contributions: (1) we investigate the effect of different surgical procedures for radical prostatectomies on a unique set of inpatient complications and (2) use a nationwide dataset comprising real world data.

Additional findings from the literature are that hospital volume was found to be more important than the type of surgical procedure according to Ploussard et al. [32], who used data from the French database on prostate removal for prostate cancer. They found that a higher annual case volume was associated with better postoperative outcomes regardless of surgical approach. These results were confirmed by another study based on French real world data by Baboudjian et al. [33] from 2023. The same study also confirmed our results, as it found laparoscopic procedures to be more favourable in terms of complication rates compared to open procedures. Baboudjian et al. also estimated that the robot-assisted form of surgery is comparatively cost-effective, as the high acquisition costs for the surgical robot are offset by a better perioperative profile and better long-term results. In contrast, a systematic review and economic modeling of the use of surgical robots in laparoscopic radical prostatectomies conducted by Ramsay et al. [6] showed that robot-assisted surgical procedures are generally more expensive than conventional surgical procedures due to high investment and maintenance costs, purporting that a high case volume should be aimed for to reduce incremental costs. The extent to which the purchase of a surgical robot is profitable for hospitals with a low case volume must be evaluated from various aspects. For example, it should be considered that robot-assisted surgical procedures are regarded as advanced and of high quality by patients, thus increasing the attractiveness of a hospital for specialists in a highly competitive market [7–9]. In addition, robot-assisted operations are less physically demanding for the surgeon [34]; therefore, cost-effectiveness studies should always be conducted from different perspectives and with varying benefit approaches to fully evaluate the complex realities and varying interests of practitioners in the healthcare sector.

## Limitations

There are limitations on the data side of this investigation. Although binding national regulations apply to data collection (coding of diagnoses and treatments), which are monitored by the supervisory authorities, errors can occur in individual cases, which can have a distorting effect on the study results. The documentation of the use of a surgical robot has no influence on the hospital’s revenue in Switzerland; it is therefore possible that the proportion of robot-assisted radical prostatectomies is underestimated in this study. Such documentation and coding limitations are common when working with administrative data, as, for instance, well researched for sepsis prevalence and incidence [35–37]. Positive and unlabeled data learning algorithms might constitute a potential solution to correct observations erroneously labelled as negative [38, 39], yet this exceeds the scope of this study.

It should also be noted that only documented and coded complications treated during the inpatient stay were considered in this study; less serious complications not requiring treatment or those detected post-surgery in an outpatient setting (as well as cases of patients dying after hospitalization) were not included. Accordingly, complication rates and risks per type of procedure could be incomplete.

In addition, disease-specific or health-related quality of life after radical prostatectomies are important factors for evaluating treatment outcomes [40, 41]; however, they could not be considered in this study. It must also be mentioned that no evidence-based risk adjustment was carried out relating to secondary diagnoses (which could be positively associated with complications).

## Conclusion

Using Swiss real-world data and a relatively large, nationwide sample, we showed that minimally invasive procedures are associated with a lower risk of procedure-related complications than open procedures for radical prostatectomies. Against this background, a (further) centralization and expansion of laparoscopic surgical techniques should be welcomed and supported; however, robot-assisted procedures do not appear to be superior to conventional laparoscopic procedures, particularly in terms of complication risk. Further (cost) effectiveness analyses from different perspectives (e.g., payer or hospital) are desirable, with real-world data used where possible.

## Electronic supplementary material

Below is the link to the electronic supplementary material.

Supplementary Material 1

Supplementary Material 2

## Data Availability

The data that support the findings of this study are available from the Swiss Federal Office of Statistics and were obtained as part of a data sharing agreement.

## Abbreviations

WHO: World Health Organization
RRP: Retropubic radical prostatectomies
LRP: Laparoscopic radical prostatectomies
RARP: Robot-assisted laparoscopic radical prostatectomies
CHOP: Swiss surgical classification
ICD: International Statistical Classification of Diseases and Related Health Problems

## References

[1] Schwendimann R, Blatter C, Dhaini S, Simon M, Ausserhofer D. The occurrence, types, consequences and preventability of in-hospital adverse events – a scoping review. BMC Health Serv Res Dezember. 2018;18(1):521. 10.1186/s12913-018-3335-z PMC6032777 29973258

[2] Fan CJ, Chien HL, Weiss MJ, He J, Wolfgang CL, Cameron JL. u. a. minimally invasive versus open surgery in the Medicare population: a comparison of post-operative and economic outcomes. Surg Endosc September. 2018;32(9):3874–80. 10.1007/s00464-018-6126-z 29484556

[3] Childers CP, Maggard-Gibbons M. Estimation of the Acquisition and operating costs for robotic surgery. JAMA 28 August. 2018;320(8):835. 10.1001/jama.2018.9219 PMC6142989 30167686

[4] Navaratnam A, Abdul-Muhsin H, Humphreys M. Updates in urologic Robot assisted surgery. F1000Research. 18. Dezember 2018;7:1948. 10.12688/f1000research.15480.1 PMC6305212 30613380

[5] Thüroff JW. Laparoskopische vs. robotische Operationen in Der Urologie. Urol Mai. 2012;51(5):615–6. 10.1007/s00120-012-2858-x 22534763

[6] Ramsay C, Pickard R, Robertson C, Close A, Vale L, Armstrong N. Systematic review and economic modelling of the relative clinical benefit and cost-effectiveness of laparoscopic surgery and robotic surgery for removal of the prostate in men with localised prostate cancer. Health Technol Assess November 2012 [zitiert 9. April 2023];16(41). Verfügbar unter: https://www.journalslibrary.nihr.ac.uk/hta/hta16410/10.3310/hta16410 PMC4780976 23127367

[7] Kuklinski D, Vogel J, Henschke C, Pross C, Geissler A. Robotic-assisted surgery for prostatectomy – does the diffusion of robotic systems contribute to treatment centralization and influence patients’ hospital choice? Health Econ Rev 10 Mai. 2023;13(1):29. 10.1186/s13561-023-00444-9 PMC10170785 37162648

[8] Aggarwal A, Lewis D, Mason M, Purushotham A, Sullivan R, Van Der Meulen J. Effect of patient choice and hospital competition on service configuration and technology adoption within cancer surgery: a national, population-based study. Lancet Oncol November. 2017;18(11):1445–53. 10.1016/S1470-2045(17)30572-7 PMC5666166 28986012

[9] Ekrutt J, Leyh-Bannurah SR, Knipper S, Schramm F, Beyer B, Maurer T. u. a. increasing the attractiveness of surgical disciplines for students: implications of a robot-assisted hands-on training course for medical education. Front Surg 21 Juli. 2022;9:953565. 10.3389/fsurg.2022.953565 PMC9349358 35937610

[10] Sung H, Ferlay J, Siegel RL, Laversanne M, Soerjomataram I, Jemal A. u. a. Global Cancer statistics 2020: GLOBOCAN estimates of incidence and Mortality Worldwide for 36 cancers in 185 countries. CA Cancer J Clin Mai. 2021;71(3):209–49. 10.3322/caac.21660 33538338

[11] Litwin MS, Tan HJ. The diagnosis and treatment of prostate Cancer: a review. JAMA 27 Juni. 2017;317(24):2532. 10.1001/jama.2017.7248 28655021

[12] Klauber J, Günster C, Gerste B, Robra BP, Schmacke N. Versorgungs-Report 2015/2016.

[13] Schrader AJ, Müller J, Janssen M, Krabbe LM. Die radikale Prostatektomie Im Wandel Der Zeit. Aktuelle Urol September. 2019;50(05):486–90. 10.1055/a-0898-3291 31141821

[14] Leow JJ, Chang SL, Meyer CP, Wang Y, Hanske J, Sammon JD. u. a. Robot-assisted Versus Open Radical Prostatectomy: a contemporary analysis of an all-payer discharge database. Eur Urol November. 2016;70(5):837–45. 10.1016/j.eururo.2016.01.044 26874806

[15] De Carlo F, Celestino F, Verri C, Masedu F, Liberati E, Di Stasi SM, Retropubic. Laparoscopic, and Robot-assisted radical prostatectomy: Surgical, Oncological, and functional outcomes: a systematic review. Urol Int. 2014;93(4):373–83. 25277444 10.1159/000366008

[16] Aron M. Robotic surgery beyond the prostate. Indian J Urol. 2014;30(3):273. 25097312 10.4103/0970-1591.135664 PMC4120213

[17] Hughes T, Rai B, Madaan S, Chedgy E, Somani B. The availability, cost, limitations, learning curve and future of Robotic systems in urology and prostate Cancer surgery. J Clin Med. März 2023;15(6):2268. 10.3390/jcm12062268 PMC10053304 36983269

[18] Moretti TBC, Magna LA, Reis LO. Surgical results and complications for Open, Laparoscopic, and Robot-assisted radical prostatectomy: a reverse systematic review. Eur Urol Open Sci Oktober. 2022;44:150–61. 10.1016/j.euros.2022.08.015 PMC9468352 36110904

[19] Sancı A, Özkaya MF, Oguz ES, Gokce Mİ, Süer E. Gülpinar O, u. a. perioperative adverse events and functional outcomes following open and robot-assisted prostatectomy in patients over age 70. Int J Clin Pract November. 2021;75(11). 10.1111/ijcp.14754. [zitiert 15. Oktober 2023];. https://onlinelibrary.wiley.com/doi/. Verfügbar unter. 10.1111/ijcp.14754 34431181

[20] Vickers AJ, Bianco FJ, Serio AM, Eastham JA, Schrag D, Klein EA. u. a. The Surgical Learning curve for prostate Cancer Control after Radical Prostatectomy. JNCI J Natl Cancer Inst 1 August. 2007;99(15):1171–7. 10.1093/jnci/djm060 17652279

[21] Leijte E, De Blaauw I, Van Workum F, Rosman C, Botden S. Robot assisted versus laparoscopic suturing learning curve in a simulated setting. Surg Endosc August. 2020;34(8):3679–89. 10.1007/s00464-019-07263-2 PMC7326898 31754849

[22] Blonde L, Khunti K, Harris SB, Meizinger C, Skolnik NS. Interpretation and impact of real-world Clinical Data for the practicing clinician. Adv Ther November. 2018;35(11):1763–74. 10.1007/s12325-018-0805-y PMC6223979 30357570

[23] Beckmann K, Garmo H, Franck Lissbrant I, Stattin P. The value of real-World Data in understanding prostate Cancer risk and improving clinical care: examples from Swedish registries. Cancers 19 Februar. 2021;13(4):875. 10.3390/cancers13040875 PMC7923148 33669624

[24] Bundesamt für Statistik (BFS). Medizinisches Kodierungshandbuch. Der offizielle Leitfaden der Kodierrichtlinien in der Schweiz. Neuchâtel 2022: Bundesamt für Statistik (BFS); 2022 [zitiert 31. Juli 2023]. (Statistik der Schweiz). Verfügbar unter: https://www.bfs.admin.ch/bfs/de/home/statistiken/gesundheit/nomenklaturen/medkk/instrumente-medizinische-kodierung.assetdetail.23446572.html

[25] Statistik Bfür. Bundesamt für Statistik. 2017 [zitiert 8. April 2023]. Schweizerische Operationsklassifikation (CHOP) - Systematisches Verzeichnis - Version 2018 | Publikation. Verfügbar unter: https://www.bfs.admin.ch/asset/de/1940914

[26] DIMDI - ICD. -10-GM Version 2018. [zitiert 8. April 2023]. Verfügbar unter: https://www.dimdi.de/static/de/klassifikationen/icd/icd-10-gm/kode-suche/htmlgm2018/

[27] Elixhauser A, Steiner C, Harris DR, Coffey RM. Januar. Comorbidity measures for Use with Administrative Data: Med Care. 1998;36(1):8–27. 10.1097/00005650-199801000-00004 9431328

[28] Sharma N, Schwendimann R, Endrich O, Ausserhofer D, Simon M. Comparing Charlson and Elixhauser comorbidity indices with different weightings to predict in-hospital mortality: an analysis of national inpatient data. BMC Health Serv Res Dezember. 2021;21(1):13. 10.1186/s12913-020-05999-5 PMC7786470 33407455

[29] SwissDRG AG. Regeln und Definitionen zur Fallabrechnung unter SwissDRG und TARPSY. 2021. Verfügbar unter: https://www.swissdrg.org/application/files/7716/3819/0804/Regeln_und_Definitionen_zur_Fallabrechnung_unter_SwissDRG_und_TARPSY.pdf

[30] Ilic D, Evans SM, Allan CA, Jung JH, Murphy D, Frydenberg M. Laparoscopic and robotic-assisted versus open radical prostatectomy for the treatment of localised prostate cancer. Cochrane Urology Group, Herausgeber. Cochrane Database Syst rev. 12. September 2017 [zitiert 5. November 2023];2017(9). Verfügbar unter: 10.1002/14651858.CD009625.pub2 10.1002/14651858.CD009625.pub2 PMC6486168 28895658

[31] Coughlin GD, Yaxley JW, Chambers SK, Occhipinti S, Samaratunga H, Zajdlewicz L. u. a. Robot-assisted laparoscopic prostatectomy versus open radical retropubic prostatectomy: 24-month outcomes from a randomised controlled study. Lancet Oncol August. 2018;19(8):1051–60. 10.1016/S1470-2045(18)30357-7 30017351

[32] Ploussard G, Grabia A, Beauval JB, Mathieu R, Brureau L, Rozet F. u. a. impact of hospital volume on postoperative outcomes after radical prostatectomy: a 5-Year nationwide database analysis. Eur Urol Focus September. 2022;8(5):1169–75. 10.1016/j.euf.2021.06.005 34147406

[33] Baboudjian M, Grabia A, Barret E, Mathieu R, Rozet F, Lequeu CE. Real-life Perioperative Outcomes of Radical Prostatectomy using the French National Registry: a Plea for Promotion of Centralized Care and Access to minimally invasive approaches. Eur Urol Oncol. Oktober 2023;S2588931123002225. 10.1016/j.euo.2023.10.006 37863772

[34] Dalsgaard T, Jensen MD, Hartwell D, Mosgaard BJ, Jørgensen A, Jensen BR. Robotic surgery is less physically demanding Than laparoscopic surgery: Paired Cross Sectional Study. Ann Surg Januar. 2020;271(1):106–13. 10.1097/SLA.0000000000002845 29923873

[35] Mellhammar L, Wollter E, Dahlberg J, Donovan B, Olséen CJ, Wiking PO. u. a. estimating Sepsis incidence using Administrative Data and Clinical Medical Record Review. JAMA Netw Open 29 August. 2023;6(8):e2331168. 10.1001/jamanetworkopen.2023.31168 PMC10466163 37642964

[36] Schwarzkopf D, Rose N, Fleischmann-Struzek C, Boden B, Dorow H, Edel A. u. a. understanding the biases to sepsis surveillance and quality assurance caused by inaccurate coding in administrative health data. Infect April. 2024;52(2):413–27. 10.1007/s15010-023-02091-y PMC10954942 37684496

[37] Rhee C, Gohil S, Klompas M. Regulatory mandates for Sepsis Care — reasons for caution. N Engl J Med Mai. 2014;370(18):1673–6. 10.1056/NEJMp1400276 PMC4718398 24738642

[38] Bekker J, Davis J. Learning from positive and unlabeled data: a survey. Mach Learn April. 2020;109(4):719–60.

[39] Vogel J, Cordier J. Application of positive and unlabeled learning: A novel approach for identifying sepsis cases from hospital administrative data. University of St.Gallen, School of Medicine, Chair of Health Economics, Policy and Management, St.Gallen; 2024 [zitiert 13. August 2024]. Verfügbar unter: https://hdl.handle.net/10419/300110

[40] Würnschimmel C, Tilki D, Huland H, Graefen M, Beyer B. Qualitätskriterien in Der Urologie: Wie Kann Eine Vergleichbarkeit Der Ergebnisse Geschaffen Werden? Urol Februar. 2021;60(2):193–8. 10.1007/s00120-020-01437-w 33439289

[41] Stranne J, Axen E, Franck-Lissbrant I, Fransson P, Frånlund M, Hugosson J. u. a. single institution followed by national implementation of systematic surgical quality control and feedback for radical prostatectomy: a 20-year journey. World J Urol Juni. 2020;38(6):1397–411. 10.1007/s00345-019-02887-4 PMC7245598 31388817

